# Signal Sensing and Transduction Are Conserved between the Periplasmic Sensory Domains of BifA and SagS

**DOI:** 10.1128/mSphere.00442-19

**Published:** 2019-07-31

**Authors:** Jozef Dingemans, Rebecca E. Al-Feghali, Holger Sondermann, Karin Sauer

**Affiliations:** a Department of Biological Sciences, Binghamton University, Binghamton, New York, USA; b Binghamton Biofilm Research Center, Binghamton University, Binghamton, New York, USA; c Department of Molecular Medicine, College of Veterinary Medicine, Cornell University, Ithaca, New York, USA; University of Maryland Medical Center

**Keywords:** BifA, HmsP, SagS, alanine substitution, biofilm drug tolerance, biofilm formation, hybrid protein, signal transduction

## Abstract

The hybrid sensor kinase SagS of Pseudomonas aeruginosa plays a key role in the transition from the planktonic to the biofilm mode of growth. Recently, we have shown that distinct sets of residues in its periplasmic HmsP sensory domain are involved in the regulation of biofilm formation or antibiotic tolerance. Interestingly, the HmsP domain of the phosphodiesterase BifA shows great predicted structural similarity to that of SagS, despite moderate sequence conservation and only a number of residues involved in SagS signaling being conserved between both proteins. Based on this observation, we hypothesized that BifA and SagS may use similar mechanisms to sense and transduce signals perceived at their periplasmic HmsP domains and, therefore, may be interchangeable. To test this hypothesis, we constructed SagS hybrids in which the HmsP domain of SagS was replaced by that of BifA (and vice versa) or by the DISMED2 sensory domain of NicD. The SagS-BifA hybrid restored attachment and biofilm formation by the Δ*bifA* mutant. Likewise, while the NicD-SagS hybrid was nonfunctional, the BifA-SagS hybrid partially restored pathways leading to biofilm formation and antibiotic tolerance in a Δ*sagS* mutant background. Furthermore, alanine substitution of key residues previously associated with the biofilm formation and antibiotic tolerance pathways of SagS impaired signal transduction by the BifA-SagS hybrid in a similar way to SagS. In conclusion, our data indicate that the nature of the sensory domain is important for proper functionality of the cytoplasmic effector domains and that signal sensing and transduction are likely conserved in SagS and BifA.

**IMPORTANCE** Biofilms have been associated with more than 60% of all recalcitrant and chronic infections and can render bacterial cells up to a thousand times more resistant to antibiotics than planktonic cells. Although it is known that the transition from the planktonic to the biofilm mode of growth involves two-component regulatory systems, increased c-di-GMP levels, and quorum sensing systems among others, the exact signaling events that lead to biofilm formation remain unknown. In the opportunistic pathogen Pseudomonas aeruginosa, the hybrid sensor kinase SagS regulates biofilm formation and antibiotic tolerance through two independent pathways via distinct residues in its periplasmic sensory domain. Interestingly, the sensory domains of SagS and BifA show great predicted structural similarity despite moderate sequence conservation. Here we show that the sensory domains of BifA and SagS are functionally interchangeable and that they use a similar mechanism of signal sensing and transduction, which broadens our understanding of how bacteria perceive and transduce signals when transitioning to the biofilm mode of growth.

## Abstract (precis)

Biofilms have been associated with more than 60% of all recalcitrant and chronic infections and can render bacterial cells up to a thousand times more resistant to antibiotics than planktonic cells. Although it is known that the transition from the planktonic to the biofilm mode of growth involves two-component regulatory systems, increased c-di-GMP levels, and quorum sensing systems among others, the exact signaling events that lead to biofilm formation remain unknown. In the opportunistic pathogen Pseudomonas aeruginosa, the hybrid sensor kinase SagS regulates biofilm formation and antibiotic tolerance through two independent pathways via distinct residues in its periplasmic sensory domain. Interestingly, the sensory domains of SagS and BifA show great predicted structural similarity despite moderate sequence conservation. Here we show that the sensory domains of BifA and SagS are functionally interchangeable and that they use a similar mechanism of signal sensing and transduction, which broadens our understanding of how bacteria perceive and transduce signals when transitioning to the biofilm mode of growth.

## INTRODUCTION

Biofilms are surface-attached, multicellular structures that are encased in a self-produced matrix of exopolysaccharides, extracellular DNA, and proteins that protect microorganisms from the actions of antibiotics as well as the immune system (1–4). Biofilm formation has been associated with more than 60% of all recalcitrant and chronic infections and poses a tremendous economic burden on health care (1, 5–8). The opportunistic pathogen Pseudomonas aeruginosa is able to cause chronic infections in burn wound patients, immunocompromised individuals, people suffering from chronic obstructive pulmonary disease (COPD), and in particular cystic fibrosis patients via the formation of biofilms (9–15). The transition from the planktonic to the biofilm lifestyle by P. aeruginosa is orchestrated via the actions of different two-component systems (TCSs) and quorum sensing (QS) systems as well as by an increase in cyclic di-GMP (c-di-GMP) levels (16–19). Among the two-component regulatory systems, the hybrid sensor SagS is a key molecular player in this transition to the sessile lifestyle of P. aeruginosa (20–22), with SagS independently controlling biofilm formation and antibiotic tolerance (22, 23). Biofilm formation was shown to be dependent on phosphotransfer between SagS and BfiS, leading to a phosphorylation cascade that involves the TCSs BfiSR, BfmSR, and MifSR (20, 22–24). In contrast, SagS affects antibiotic tolerance of biofilms by indirectly regulating the c-di-GMP-responsive transcriptional regulator BrlR, which activates the expression of an array of multidrug efflux pumps and ABC transporters (22, 23, 25–30). SagS is composed of an N-terminal periplasmic sensory domain (HmsP), a histidine kinase domain (HisKA), and a C-terminal phosphoreceiver (Rec) domain (20, 22, 23). The HmsP domain was originally identified in the Yersinia pestis biofilm regulatory protein HmsP. HmsP harbors in addition to the sensory HmsP domain a HAMP domain, EAL domain, and GGDEF domain, with the HmsP protein demonstrating phosphodiesterase (PDE) activity (31, 32). While homologs of the biofilm regulatory protein HmsP have since been identified in Salmonella enterica serovar Typhimurium and Escherichia coli, no homologs of the HmsP protein have been identified in the genome of P. aeruginosa. Instead, two P. aeruginosa proteins harboring the HmsP domain have been identified as SagS and BifA. Using sequence alignments, we previously demonstrated that the periplasmic sensory domains of SagS and BifA from P. aeruginosa and the periplasmic sensory domain of HmsP (Yersinia pestis) show considerable homology at the amino acid sequence level (23).

Two distinct sets of amino acids in the periplasmic sensory domain of SagS contribute to SagS modulation of biofilm formation or antibiotic tolerance, with amino acid residues L154 and D105 being key to biofilm formation and drug tolerance, respectively (23). Furthermore, interference with the biofilm formation or antibiotic tolerance pathways significantly affected P. aeruginosa virulence during chronic but not acute infections, with mutation of residue L154, but not D105, leading to a significant reduction in bacterial load in a murine chronic pneumonia model, while mutation of D105, but not L154, affected tolerance to tobramycin in the same chronic infection model (33).

Similar to SagS, the P. aeruginosa inner membrane protein BifA harbors a periplasmic sensory HmsP domain (23). In addition, BifA harbors both GGDEF and EAL domains. While BifA has only been shown to have phosphodiesterase activity (34), mutational analyses of conserved residues showed that both the GGDEF and EAL domains contribute to the phosphodiesterase activity of the protein (34). BifA inversely regulates biofilm formation and motility by P. aeruginosa PA14 and two strains belonging to the Pseudomonas syringae complex, with BifA negatively regulating biofilm formation and positively regulating motility (34–36). In contrast, BifA was shown to suppress biofilm formation without affecting motility by Pseudomonas putida (37). Virulence assays in olive and tomato, furthermore, indicated that BifA contributes to the fitness and virulence of bacterial strains belonging to the Pseudomonas syringae complex (36). Recently, it was also shown that manganese (Mn^2+^) and hydrogen peroxide (H_2_O_2_) have an effect on *bifA* expression, with higher concentrations of Mn^2+^ negatively impacting biofilm formation (coinciding with an upregulation of *bifA*) and lower levels of H_2_O_2_ enhancing biofilm formation (coinciding with a downregulation of *bifA*) in P. putida (38). Despite SagS and BifA both sharing an HmsP domain, however, the two proteins have little in common. SagS and BifA contribute to distinct pathways that have an opposite effect on biofilm formation, with SagS positively regulating biofilm formation, while BifA represses biofilm formation. What they have in common is that similar to SagS, the signal sensed by BifA and how it is transmitted to its catalytic domain remain unknown, and both proteins trigger a switch in the bacterial lifestyle. In an effort to determine whether both proteins rely on a similar signal relay to affect biofilm formation, we aimed to determine in this study whether the periplasmic sensory domains of BifA and SagS are functionally interchangeable, despite their disparate functions in biofilm formation and drug tolerance. To test our hypothesis, we made use of hybrid proteins in which the periplasmic sensory domains of SagS and BifA were exchanged.

## RESULTS

### The sensory domains of BifA and SagS are structurally similar.

**FIG 1:**
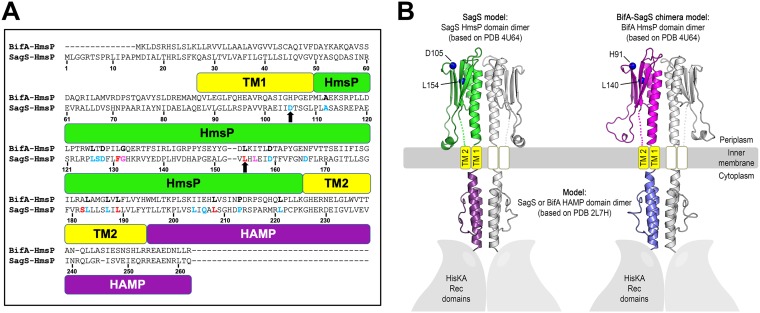
Sequence alignment and predictive structural models of the HmsP and HAMP domains of SagS and BifA-SagS. (A) Sequence alignment of the sensory HmsP domains of BifA and SagS. Based on structural predictions (secondary structure) using the PHYREII server, the periplasmic sensory domain of SagS is shown in green, the transmembrane helices in yellow, and the HAMP domain in purple. Residues previously found to be involved in SagS regulating biofilm formation (cyan), antibiotic tolerance (red), or both phenotypes (magenta) are highlighted in the HmsP-SagS sequence, with the key residues D105A and L154 indicated by black arrows. Residues conserved between SagS and BifA are highlighted in bold (black) in the HmsP-BifA sequence. (B) Side-by-side comparison of the predictive structural models of the periplasmic sensory domains of SagS (green) and BifA (pink) and their HAMP domains (purple for SagS, blue for BifA). Structural predictions were initially carried out using the PHYREII server, identifying high-confidence target structures and alignments for subsequent homology modeling of domain dimers. Amino acid residues (blue spheres marking C-α positions) that were previously shown to be involved in SagS regulating biofilm formation (L154) and antibiotic tolerance (D105) and their corresponding residues in BifA (L140 and H91, respectively) were mapped onto the three-dimensional models.

Similar to P. aeruginosa BifA, SagS harbors a periplasmic sensory HmsP domain. However, the sequences are not identical, which is apparent by the HmsP domains of BifA and SagS only sharing 29.3% sequence identity and 51.3% similarity based on pairwise sequence alignment (see Fig. S1 in the supplemental material). Moreover, only 3 out of 5 key residues contributing to SagS regulating attachment or 8 out of 13 key residues contributing to antibiotic tolerance are conserved between these two domains (Fig. 1A; Fig. S1). Despite the apparent differences, their predicted structural similarity is remarkable (Fig. 1B). As is the case for the sensory domain of SagS, the HmsP domain of BifA harbors a predicted beta-sheet formed by four antiparallel beta strands, which has been identified as a ligand-binding site in other sensory proteins (39). Furthermore, a small helical motif that is predicted to be closely associated with the inner membrane in SagS (23) is also present in BifA (Fig. 1B). Interestingly, the L154 and D105 residues in the HmsP domain of SagS, which have previously been shown to contribute to biofilm formation or antibiotic tolerance, are, respectively, located in the aforementioned beta-sheet and a turn between two beta strands that are conserved between BifA and SagS (Fig. 1). Based on the predicted structural similarity, we hypothesize that the sensory domains of BifA and SagS function in a similar way.

10.1128/mSphere.00442-19.1 FIG S1 Pairwise sequence identity and similarity between the HmsP domains of BifA (top) and SagS (bottom). A global alignment was performed for the BifA and SagS HmsP sequences using the EMBOSS Needle Global Alignment tool on the EMBL-EBI website (https://www.ebi.ac.uk/Tools/psa/emboss_needle). Identical, similar, and nonidentical residues in the sequence alignment are indicated by vertical stripes, double dots, and single dots, respectively. Download FIG S1, PDF file, 0.2 MB. Copyright © 2019 Dingemans et al. 2019 Dingemans et al. This content is distributed under the terms of the Creative Commons Attribution 4.0 International license.

### Replacement of the sensory domain of SagS or BifA does not affect protein stability.

**FIG 2:**
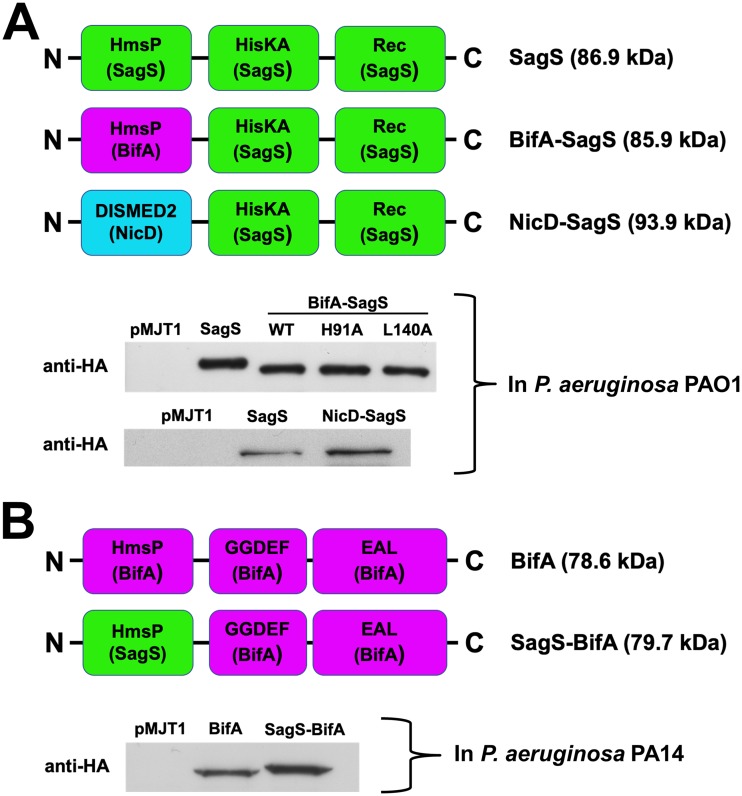
Domain organization and protein levels of wild-type SagS and BifA as well as the hybrid constructs. (A) Overview of the SagS, BifA-SagS, and NicD-SagS constructs. The BifA-SagS hybrid contains the N-terminal HmsP domain of BifA comprising residues 1 to 248 (purple), fused to the histidine kinase (HisKA) and phosphoreceiver (Rec) domains of SagS (starting at position 263 of SagS, indicated in green), resulting in a predicted HA-tagged protein product that is 1 kDa smaller than wild-type SagS. NicD-SagS contains the DISMED2 sensory domain and first transmembrane helix of NicD comprising residues 1 to 254 (blue), fused to the HisKA and phosphoreceiver domains of SagS via the HAMP domain (starting at position 195 of SagS, indicated in green). Representative immunoblots demonstrate production of SagS, BifA-SagS, and BifA-SagS variants (upper blot) and NicD-SagS (lower blot) in P. aeruginosa PAO1 strains inactivated in *sagS*, grown planktonically to exponential phase. A Δ*sagS* mutant strain harboring the empty plasmid pMJT1 was used as a control. A total of 15 μg total cell extract was loaded. The immunoblots were probed for the presence of SagS, NicD-SagS, BifA-SagS, or variants using anti-HA antibodies. (B) Overview of the BifA and SagS-BifA constructs. The SagS-BifA hybrid contains the N-terminal HmsP domain of SagS comprising residues 1 to 262 (green) fused to the GGDEF/EAL domains of BifA (starting at position 249 of BifA, indicated in purple). The representative immunoblot demonstrates the presence of BifA and SagS-BifA in total cell extracts obtained from P. aeruginosa PA14 strains inactivated in *bifA* overexpressing *bifA* or *sagS*-*bifA*, grown planktonically to exponential phase. The Δ*bifA* mutant strain harboring the empty plasmid pMJT1 was used as control. A total of 30 μg total cell extract was loaded. The immunoblots were probed for the presence of BifA or SagS-BifA using anti-HA antibodies.

To test whether the sensory domains of BifA and SagS are functionally similar, we created a hybrid protein, referred to as BifA-SagS, by replacing the N-terminal HmsP domain of SagS (including the transmembrane and HAMP motifs) with that of BifA, while maintaining the histidine kinase and phosphoreceiver domains of SagS (Fig. 2A). In addition, we generated two additional chimeric proteins. For one, we constructed a SagS hybrid that carried a periplasmic domain other than the HmsP domain. We chose the periplasmic DISMED2 sensory domain of the diguanylate cyclase (DGC) NicD, which has previously been demonstrated to be involved in glutamate sensing (40). The hybrid protein, referred to as NicD-SagS, was constructed by fusing the DISMED2 domain and first transmembrane helix of the diguanylate cyclase NicD to the HAMP domain of SagS (Fig. 2A). Since the DISMED2 domain of NicD has a different fold than the HmsP domain of BifA or SagS (39), this hybrid protein served as a control to determine whether any sensory domain would be able to restore the functionality of SagS. The second hybrid protein, referred to as SagS-BifA, was generated to determine whether the HmsP domain of BifA can be functionally substituted for by the HmsP domain of SagS without affecting the phosphodiesterase activity of BifA in P. aeruginosa PA14 (34). The SagS-BifA hybrid was constructed by substituting for the HmsP domain of BifA with that of SagS (Fig. 2B). As we aimed at using the hybrid in a P. aeruginosa PA14 background, the hybrid was constructed using genomic DNA obtained from P. aeruginosa PA14. It is of interest to note that with the exception of one amino acid that is not critical for the toggle switch function of SagS, the HmsP domains of SagS are identical in PAO1 and PA14 (see Fig. S2 in the supplemental material). Likewise, the amino acid sequences of the HmsP domains of BifA are identical in PAO1 and PA14 (see Fig. S3 in the supplemental material).

10.1128/mSphere.00442-19.2 FIG S2 Pairwise sequence identity and similarity between the HmsP domains of SagS in PA14 and PAO1. A global alignment was performed for the SagS HmsP sequences of PA14 (top) and PAO1 (bottom) using the EMBOSS Needle Global Alignment tool on the EMBL-EBI website (https://www.ebi.ac.uk/Tools/psa/emboss_needle). Identical, similar, and nonidentical residues in the sequence alignment are indicated by vertical stripes, double dots, and single dots, respectively. Residues previously found to be involved in SagS regulating biofilm formation (cyan), antibiotic tolerance (red), or both phenotypes (magenta) are highlighted in the HmsP-SagS sequence of PAO1, with the key residues D105A and L154 indicated by black arrows. Download FIG S2, PDF file, 0.1 MB. Copyright © 2019 Dingemans et al. 2019 Dingemans et al. This content is distributed under the terms of the Creative Commons Attribution 4.0 International license.

10.1128/mSphere.00442-19.3 FIG S3 Pairwise sequence identity and similarity between the HmsP domains of BifA in PA14 and PAO1. A global alignment was performed for the SagS HmsP sequences of PA14 (top) and PAO1 (bottom) using the EMBOSS Needle Global Alignment tool on the EMBL-EBI website (https://www.ebi.ac.uk/Tools/psa/emboss_needle). Identical, similar, and nonidentical residues in the sequence alignment are indicated by vertical stripes, double dots, and single dots, respectively. Download FIG S3, PDF file, 0.1 MB. Copyright © 2019 Dingemans et al. 2019 Dingemans et al. This content is distributed under the terms of the Creative Commons Attribution 4.0 International license.

To determine whether the hybrid proteins are produced in a stable and soluble manner, the respective proteins were overproduced under planktonic conditions and their production levels in clarified total cell extract subsequently analyzed via immunoblot analysis using an antihemagglutinin (anti-HA) antibody. Similar protein levels were observed for BifA-SagS, NicD-SagS, and wild-type SagS in PAO1 (Fig. 2A) as well as for SagS-BifA compared to wild-type BifA in PA14 (Fig. 2B), indicating that protein stability was not affected by replacing the sensory domains.

### Interchanging the HmsP domains of SagS and BifA leads to restoration of attachment and biofilm formation in both Δ*sagS* and Δ*bifA* mutant backgrounds.

**FIG 3:**
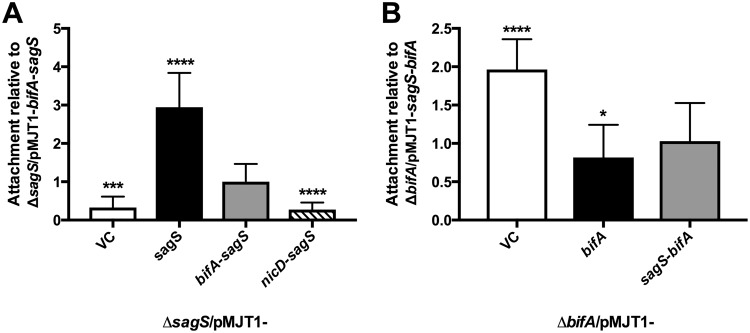
Attachment levels of the Δ*sagS* mutant complemented with *sagS* or hybrid constructs and the *ΔbifA* mutant complemented with *bifA* or the *sagS-bifA* hybrid. Attachment was determined via crystal violet staining following 24 h of growth under shaking conditions. (A) Attachment of P. aeruginosa PAO1 strains inactivated in *sagS* that either harbor the empty vector pMJT1 or express *sagS*, *bifA-sagS*, and *nicD-sagS*. Attachment is shown relative to the Δ*sagS*/pMJT1-*bifA*-*sagS* strain. (B) Attachment of P. aeruginosa PA14 strains inactivated in *bifA* that either harbor the empty vector pMJT1 or express *bifA* or *sagS-bifA*. Attachment is shown relative to the Δ*bifA*/pMJT1-*sagS*-*bifA* strain (B). All assays were repeated at least three times, with each repeat consisting of at least 8 technical replicates. Error bars denote standard deviation. * (*P* < 0.05), *** (*P* < 0.001), and **** (*P* < 0.0001) indicate significantly different from the Δ*sagS*/pMJT1-*bifA*-*sagS* or Δ*bifA*/pMJT1-*sagS*-*bifA* strain. NS, not significantly different from the Δ*sagS*/pMJT1-*bifA*-*sagS* or Δ*bifA*/pMJT1-*sagS*-*bifA* strain.

Previous findings indicated SagS to contribute to attachment (20, 22, 23). The finding of Δ*sagS* mutant strains being impaired in attachment after 24 h of growth relative to wild-type P. aeruginosa (20, 23) allowed us to test whether complementation of the Δ*sagS* mutation with the *bifA*-*sagS* hybrid gene could restore the attachment phenotype to wild-type levels. The Δ*sagS* strain complemented with *bifA*-*sagS* showed intermediate levels of attachment that were approximately 3-fold higher than those of the Δ*sagS*/pMJT1 strain but approximately 3-fold lower than the Δ*sagS* mutant complemented with wild-type *sagS* (Fig. 3A). In contrast, overexpression of *nicD*-*sagS* in Δ*sagS* had no effect on attachment, which is apparent by the attachment being similar to that in the Δ*sagS* mutant harboring the empty vector (Fig. 3A).

**FIG 4:**
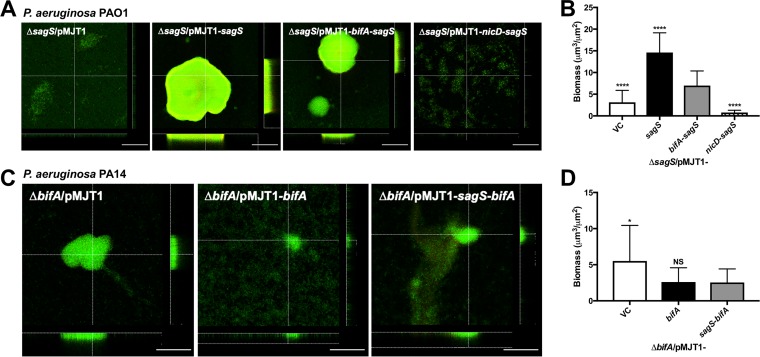
Biofilm formation of the Δ*sagS* mutant complemented with *sagS* or hybrid constructs and the *ΔbifA* mutant complemented with *bifA* or the *sagS-bifA* hybrid. (A) Representative confocal images showing the architecture of biofilms formed by P. aeruginosa PAO1 *ΔsagS*/pMJT1, *ΔsagS*/pMJT1-*sagS*, *ΔsagS*/pMJT1-*bifA*-*sagS*, and *ΔsagS*/pMJT1-*nicD*-*sagS*. Confocal images were obtained after 3 days of growth in 5-fold-diluted LB medium. Biofilms were stained with the LIVE/DEAD BacLight viability stain (Life Technologies). White bars = 100 μm. (B) Total biomass formed by the respective *ΔsagS* mutant strains after 3 days of growth, as determined using confocal images and subsequent COMSTAT analysis. (C) Representative confocal images of biofilms formed by P. aeruginosa PA14 *ΔbifA*/pMJT1, *ΔbifA*/pMJT1-*bifA*, and *ΔbifA*/pMJT1-*sagS*-*bifA*. Confocal images were obtained after 3 days of growth in 5-fold-diluted LB medium. Biofilms were stained with the LIVE/DEAD BacLight viability stain (Life Technologies). White bars = 100 μm. (D) Total biomass formed by the respective *ΔbifA* mutant strains after 3 days of growth, as determined using confocal images and subsequent COMSTAT analysis. All assays were repeated at least two times, with a minimum of 6 images being acquired. Error bars indicate standard deviation. * (*P* < 0.05) and **** (*P* < 0.0001) indicate significantly different from the Δ*sagS*/pMJT1-*bifA*-*sagS* or Δ*bifA*/pMJT1-*sagS*-*bifA* strain. NS, not significantly different from the Δ*sagS*/pMJT1-*bifA*-*sagS* or Δ*bifA*/pMJT1-*sagS*-*bifA* strain.

We likewise determined whether the BifA-SagS hybrid is capable of substitution for SagS with respect to biofilm formation. Biofilms formed by the Δ*sagS* mutant have been characterized as being unstructured and mainly comprised of a monolayer of cells (20, 22, 23). This was furthermore supported by Δ*sagS* biofilms being characterized by a low biofilm biomass relative to wild-type P. aeruginosa (20, 22, 23). Multicopy expression of *bifA*-*sagS* in the Δ*sagS* mutant led to the formation of biofilms characterized by the presence of microcolonies. Based on visual inspection, these microcolonies were considerably larger than those formed by the Δ*sagS*/pMJT1 strain but smaller than those formed by the Δ*sagS* strain expressing wild-type *sagS* (Fig. 4A and B). Not surprisingly, the total biomass for the Δ*sagS*/pMJT1-*bifA*-*sagS* strain was significantly higher (∼2-fold) than that observed for the Δ*sagS*/pMJT1 strain but significantly lower (∼2-fold) than the biomass formed by the Δ*sagS*/pMJT1-*sagS* strain (Fig. 4A and B). Similarly, the thickness of biofilms by Δ*sagS*/pMJT1-*bifA*-*sagS* was intermediary to that of biofilms formed by the Δ*sagS*/pMJT1 strain and the Δ*sagS* strain expressing wild-type *sagS* (see Fig. S4 in the supplemental material). In contrast, biofilms by the Δ*sagS*/pMJT1-*nicD*-*sagS* strain were comparable to Δ*sagS* biofilms (Fig. 4A and B; Fig. S4). Our findings so far suggested that the BifA-SagS hybrid is partially functional in restoring attachment and biofilm formation by the Δ*sagS* mutant to wild-type levels, while a SagS hybrid harboring a periplasmic domain other than the HmsP domain is not capable of substituting for SagS.

10.1128/mSphere.00442-19.4 FIG S4 Thickness of biofilms formed by Δ*sagS* mutant strains expressing *sagS* or *sagS* hybrid constructs *bifA-sagS* and *nicD*-*sagS*. Average (A) and maximum (B) biofilm thickness were determined via COMSTAT analysis of confocal images obtained after 3 days of growth of the Δ*sagS*/pMJT1, Δ*sagS*/pMJT1-*sagS*, *ΔsagS*/pMJT1-*bifA*-*sagS*, and *ΔsagS*/pMJT1-*nicD*-*sagS* strains. Experiments were repeated at least three times, with a minimum of 6 images being acquired. Error bars indicate standard deviation. **** (*P* < 0.0001) indicates significantly different from the Δ*sagS*/pMJT1-*bifA*-*sagS* strain. NS, not significantly different from the Δ*sagS*/pMJT1-*bifA*-*sagS* strain. Download FIG S4, PDF file, 0.06 MB. Copyright © 2019 Dingemans et al. 2019 Dingemans et al. This content is distributed under the terms of the Creative Commons Attribution 4.0 International license.

To further determine whether the HmsP domains of SagS and BifA are interchangeable, we next tested attachment and biofilm formation by strains expressing the SagS-BifA hybrid. Previous findings demonstrated that inactivation of the Δ*bifA* mutant coincides with significantly enhanced attachment compared to the parental strain, P. aeruginosa PA14 (34). We made use of this finding to determine whether complementation of Δ*bifA* in P. aeruginosa PA14 with *sagS*-*bifA* restored attachment to wild-type levels. Relative to Δ*bifA*, multicopy expression of *sagS*-*bifA* significantly decreased attachment (∼2-fold). Moreover, multicopy expression of *sagS*-*bifA* resulted in an overall similar decrease in attachment to multicopy expression of *bifA* in the Δ*bifA* mutant (Fig. 3B). To further explore the functional similarity between BifA and SagS-BifA, we next evaluated biofilm formation. In agreement with previous findings (34), multicopy expression of *bifA* in the Δ*bifA* mutant resulted in significantly reduced biofilm biomass accumulation compared to the Δ*bifA*/pMJT1 strain (Fig. 4C and D). Likewise, multicopy expression of *sagS*-*bifA* also resulted in significantly reduced biofilm biomass accumulation, with biofilms by the Δ*bifA*/pMJT1-sagS-*bifA* strain being similar in appearance to biofilms by the Δ*bifA*/pMJT1-*bifA* strain (Fig. 4C and D). It is thus not surprising that biofilm biomass (Fig. 4D) and thickness (see Fig. S5 in the supplemental material) were significantly lower for the Δ*bifA*/pMJT1-*sagS*-*bifA* strain compared to the Δ*bifA*/pMJT1 strain, but not statistically different from the Δ*bifA*/pMJT1-*bifA* strain.

10.1128/mSphere.00442-19.5 FIG S5 Thickness of biofilms formed by Δ*bifA* mutant strains expressing *bifA* or *sagS*-*bifA*. Average (A) and maximum (B) biofilm thickness were determined via COMSTAT analysis of confocal images obtained after 3 days of growth of the Δ*bifA*/pMJT1, Δ*bifA*/pMJT1-*bifA*, and *ΔbifA*/pMJT1-*sagS*-*bifA* strains. Experiments were repeated at least three times, with a minimum of 6 images being acquired. Error bars indicate standard deviation. ** (*P* < 0.01) indicates significantly different from the Δ*bifA*/pMJT1-*sagS*-*bifA* strain. NS, not significantly different from the Δ*bifA*/pMJT1-*sagS*-*bifA* strain. Download FIG S5, PDF file, 0.05 MB. Copyright © 2019 Dingemans et al. 2019 Dingemans et al. This content is distributed under the terms of the Creative Commons Attribution 4.0 International license.

### Complementation of Δ*sagS* with *bifA*-*sagS* results in intermediate levels of BfiS phosphorylation.

**FIG 5:**
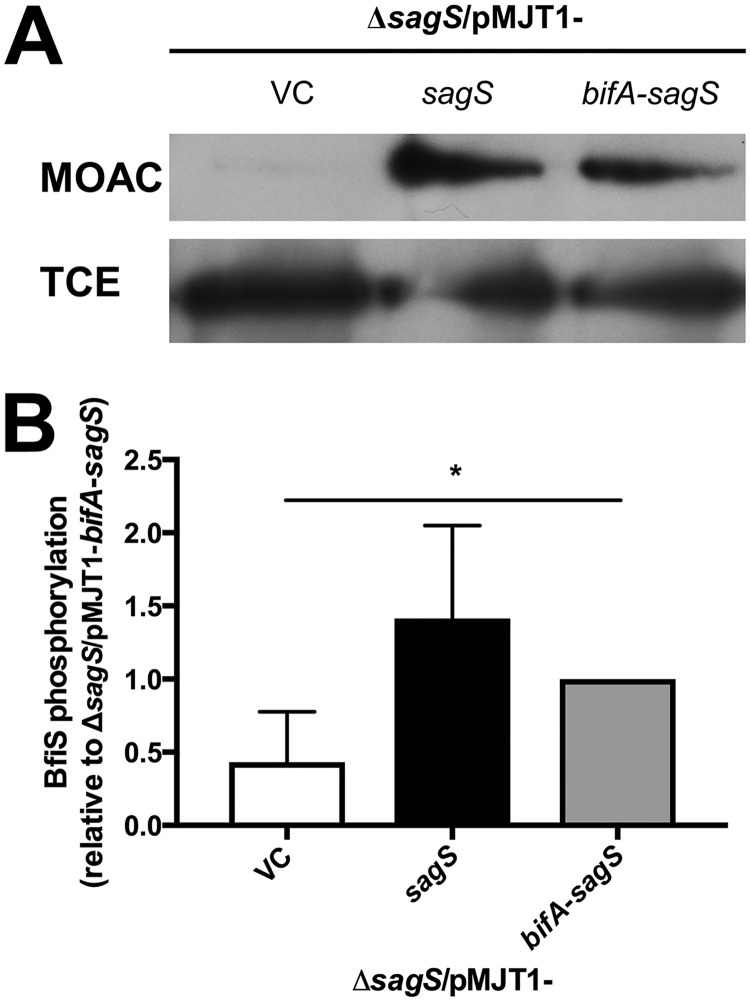
BfiS phosphorylation by the Δ*sagS*/pMJT1, Δ*sagS*/pMJT1-*sagS*, and Δ*sagS*/pMJT1-*bifA-sagS* strains under biofilm conditions. (A) Detection of BfiS in total cell extracts (TCE) and in MOAC-enriched phosphoproteomes (MOAC) of P. aeruginosa Δ*sagS* cells harboring the empty vector pMJT1 or overexpressing wild-type *sagS* or the *bifA*-*sagS* hybrid. Cell-free total cell extracts were obtained from 3-day-old biofilms of Δ*sagS*/pMJT1, Δ*sagS/*pMJT1*-sagS*, and *ΔsagS*/pMJT1-*bifA*-*sagS* cells. For MOAC samples, the entire MOAC eluate concentrated using methanol-chloroform precipitation was loaded. Representative images are shown. (B) Quantification of BfiS phosphorylation, based on relative intensity of protein bands detectable following probing for BfiS with anti-V5 antibodies and subsequent analysis using ImageJ (58). Experiments were carried out in triplicate. Error bars denote standard deviation. *, significantly different (*P* ≤ 0.05), as determined by ANOVA and PrismV.

Considering that the hybrid proteins BifA-SagS and SagS-BifA partly restored attachment and biofilm formation in a manner similar to SagS and BifA, respectively, we next asked whether the BifA-SagS hybrid contributes to the toggle switch function of SagS in a manner similar to SagS. Biofilm formation by P. aeruginosa has been shown to be dependent on phosphotransfer between SagS and BfiS (20, 23). Considering that expression of the *bifA-sagS* hybrid partly restored biofilm formation, we surmised that similar to SagS, the BifA-SagS hybrid is likewise capable of phosphorylating BfiS. We therefore determined the phosphorylation status of BfiS by first purifying phosphorylated proteins via metal-oxide affinity chromatography (MOAC) and subsequently visualizing BfiS by immunoblot analysis. In support of the biofilm formation phenotype, immunoblot analysis revealed intermediate levels of phosphorylated BfiS for the BifA-SagS hybrid that were >2-fold higher than those observed for the Δ*sagS*/pMJT1 strain but lower than those for the Δ*sagS*/pMJT1-*sagS* strain (Fig. 5).

### Antibiotic tolerance is restored to nearly wild-type levels in a Δ*sagS* mutant complemented with *bifA*-sag*S*.

**FIG 6:**
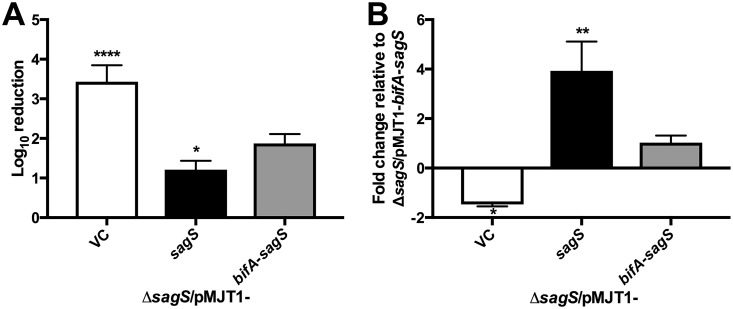
The Δ*sagS*/pMJT1-*bifA-sagS* strain displays intermediate tobramycin susceptibility and *brlR* expression levels compared to the Δ*sagS*/pMJT1 and Δ*sagS*/pMJT1-*sagS* strains. (A) Tobramycin susceptibility of 2-day-old biofilms formed by Δ*sagS/*pMJT1, Δ*sagS*/pMJT1*-sagS*, and *ΔsagS*/pMJT1-*bifA*-*sagS* cells. Viability was determined via CFU counts. Susceptibility is expressed as log_10_ reduction. Experiments were carried out in triplicate. Error bars denote standard deviation. (B) *brlR* expression levels of the Δ*sagS/*pMJT1, Δ*sagS*/pMJT1*-sagS*, and *ΔsagS*/pMJT1-*bifA*-*sagS* strains relative to the *ΔsagS*/pMJT1-*bifA*-*sagS* strain. For gene expression analysis by qRT-PCR, RNA obtained from 3-day-old biofilms was used. The *mreB* gene was selected as the housekeeping gene. Experiments were at least carried out in triplicate. Error bars indicate standard deviation. * (*P* < 0.05), ** (*P* < 0.01), and **** (*P* < 0.0001) indicate significantly different from the Δ*sagS*/pMJT1-*bifA*-*sagS* strain. NS, not significantly different from the Δ*sagS*/pMJT1-*bifA*-*sagS* strain.

In addition to being involved in biofilm formation, SagS also contributes to antibiotic tolerance of biofilms, via the indirect regulation of the transcriptional regulator BrlR, which regulates several multidrug efflux pumps and ABC transporters (22, 23, 27–30). Therefore, we tested if the BifA-SagS hybrid was able to restore antibiotic tolerance of biofilms by Δ*sagS* mutant strains. Biofilms formed by the Δ*sagS*/pMJT1-*bifA*-*sagS* strain were significantly less susceptible to tobramycin compared to those of the Δ*sagS*/pMJT1 strain (1.9-log_10_ reduction versus a 3.4-log_10_ reduction) but more susceptible than biofilms formed by the Δ*sagS*/pMJT1-*sagS* strain (1.9-log_10_ reduction versus 1.2-log_10_ reduction) (Fig. 6A). Given that expression of *bifA-sagS* partly restored the susceptibility phenotype of Δ*sagS* mutant biofilms, we furthermore surmised that the intermediate tobramycin susceptibility levels observed for Δ*sagS* strains expressing *bifA*-*sagS* relative to the Δ*sagS* and Δ*sagS*/pMJT1*-sagS* strains are due to intermediate *brlR* expression levels. In support of this hypothesis, the *brlR* expression levels detected for the Δ*sagS*/pMJT1-*bifA*-*sagS* strain were significantly higher than those observed for the Δ*sagS*/pMJT1 strain and significantly lower than those observed for the Δ*sagS*/pMJT1-*sagS* strain (Fig. 6B).

### Residues involved in biofilm formation or antibiotic tolerance are functionally conserved between BifA and SagS.

Our findings so far demonstrated that the BifA-SagS hybrid can partly restore the biofilm formation and antibiotic tolerance phenotypes by Δ*sagS* mutant strains to near wild-type levels. These findings led us to conclude that the periplasmic sensory domains of BifA and SagS are partly interchangeable, despite the two proteins contributing to different pathways that have an opposite effect on biofilm formation and tolerance. To provide further evidence of the interchangeability of the two domains, we next focused on two residues that have previously been identified to be key to SagS regulation of biofilm formation or antibiotic tolerance: SagS residues D105 and L154 (23, 33).

It is of interest to note that the SagS-L154 residue involved in biofilm formation is conserved in BifA (L140), while the residue SagS-D105, involved in antibiotic tolerance, is not (Fig. 1A). Instead, pairwise alignments suggested a histidine residue in the BifA sequence (see H91 in BifA). However, both residues are located in regions that are predicted to be structurally conserved in BifA and SagS (Fig. 1). The two residues of interest, BifA-H91 and BifA-L140, were subjected to site-directed mutagenesis to investigate the effect of alanine substitution of the BifA residues L140 and H91 on the biofilm formation and antibiotic tolerance phenotypes of the BifA-SagS hybrid. The resulting hybrid variants are referred to as BifA-SagS_H91A and BifA-SagS_L140A.

**FIG 7:**
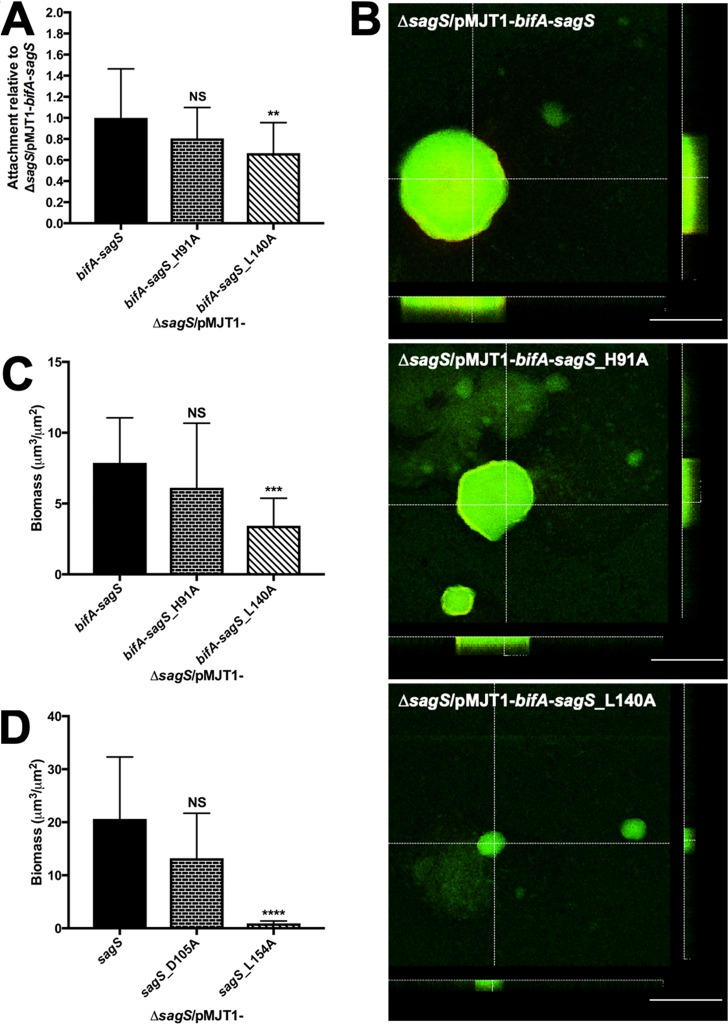
Effect of mutation of H91A and L140 on attachment and biofilm formation. (A) Attachment of the indicated *ΔsagS* mutant strains was determined via crystal violet staining following 24 h of growth under shaking conditions. Attachment is expressed relative to the Δ*sagS*/pMJT1-*bifA*-*sagS* strain. Experiments were repeated at least three times, with each repeat consisting of at least 8 technical replicates. Error bars denote standard deviation. ** (*P* < 0.01) indicates significantly different from the Δ*sagS*/pMJT1-*bifA*-*sagS* strain. NS, not significantly different from the Δ*sagS*/pMJT1-*bifA*-*sagS* strain. (B) Representative confocal images showing the architecture of biofilms formed by the P. aeruginosa *ΔsagS*/pMJT1-*bifA*-*sagS*, *ΔsagS*/pMJT1-*bifA*-*sagS*_H91A, and *ΔsagS*/pMJT1-*bifA*-*sagS*_L140A strains. Confocal images were obtained after 3 days of growth in 5-fold-diluted LB medium. Biofilms were stained with the LIVE/DEAD BacLight viability stain (Life Technologies). White bars = 100 μm. (C) Total biomass of biofilms formed by indicated *ΔsagS* mutant strains, as determined using confocal images and subsequent COMSTAT analysis. Assays were repeated at least three times, with a minimum of 6 images being acquired. Error bars indicate standard deviation. *** (*P* < 0.001) indicates significantly different from the Δ*sagS*/pMJT1-*bifA*-*sagS* strain. NS, not significantly different from the Δ*sagS*/pMJT1-*bifA*-*sagS* strain. (D) Total biomass of biofilms formed by the *ΔsagS*/pMJT1-*sagS*, *ΔsagS*/pMJT1-*sagS*_D105, and *ΔsagS*/pMJT1-*sagS*_L154A strains after 3 days of growth, as determined using confocal images and subsequent COMSTAT analysis. Assays were repeated at least three times, with a minimum of 6 images being acquired. Error bars indicate standard deviation. **** (*P* < 0.001) indicates significantly different from the Δ*sagS*/pMJT1-*sagS* strain. NS, not significantly different from the Δ*sagS*/pMJT1-*sagS* strain.

Relative to Δ*sagS* mutant strains expressing *bifA-sagS*, expression of the *bifA-sagS*_L140A variant correlated with a significant decrease in attachment (Fig. 7A), biofilm biomass accumulation (Fig. 7B and C), and an overall decrease in the thickness of biofilms (Fig. 7B; see Fig. S6 in the supplemental material). In contrast, alanine substitution of BifA-H91 had no apparent effect on attachment or the biofilm architecture (Fig. 7A to C). However, an apparent reduction in the biofilm thickness was noted in Δ*sagS* mutant strains expressing the *bifA-sagS*_H91A variant relative to strains expressing *bifA-sagS* (Fig. S6).

10.1128/mSphere.00442-19.6 FIG S6 Thickness of biofilms formed by Δ*sagS* mutant strains expressing *bifA-sagS* or the *bifA*-*sagS_*91A and *bifA*-*sagS*_L140A variant constructs. Average (A) and maximum (B) thickness of biofilms formed by the *ΔsagS*/pMJT1-*bifA*-*sagS* strain compared to the *ΔsagS*/pMJT1-*bifA*-*sagS*_H91A and *ΔsagS*/pMJT1-*bifA*-*sagS*_L140A strains after 3 days of growth as determined using confocal images and subsequent COMSTAT analysis. All assays were repeated at least three times, with a minimum of 6 images being acquired. Error bars indicate standard deviation. * (*P* < 0.05), *** (*P* < 0.001), and **** (*P* < 0.0001) indicate significantly different from the Δ*sagS*/pMJT1-*bifA*-*sagS* strain. NS, not significantly different from the Δ*sagS*/pMJT1-*bifA*-*sagS* strain. Download FIG S6, PDF file, 0.09 MB. Copyright © 2019 Dingemans et al. 2019 Dingemans et al. This content is distributed under the terms of the Creative Commons Attribution 4.0 International license.

It is of interest to note that in agreement with previous findings, alanine substitution of the two key residues of SagS, D105 and L154, had a similar effect on biofilm biomass accumulation as substitution of the BifA residues H91 and L140 (Fig. 7D; see Fig. S7 in the supplemental material). Considering that residue SagS-L154, but not SagS-D105, has previously been shown to contribute to attachment and biofilm formation (23, 33), our findings suggest that like residue SagS-L154, residue L140 present in the BifA-SagS hybrid serves a similar function. Moreover, alanine substitution of H91 had little to no effect on BifA-SagS function with respect to attachment and biofilm formation.

10.1128/mSphere.00442-19.7 FIG S7 Comparison of (A) attachment and (B) biofilm biomass by the Δ*sagS*/pMJT1-*sagS* strain and its D105A and L154A variants and the Δ*sagS*/pMJT1-*bifA-sagS* strain and its H91A and L140A variants. (A) Attachment was determined via crystal violet staining following 24 h of growth under shaking conditions. Attachment is expressed relative to the Δ*sagS*/pMJT1-*sagS* strain (black) or to the *ΔsagS*/pMJT1-*bifA*-*sagS* strain (gray). All assays were repeated at least three times, with each repeat consisting of at least 8 technical replicates. Error bars denote standard deviation. (B) Total biomass of biofilms formed by the *ΔsagS*/pMJT1-*sagS*, *ΔsagS*/pMJT1-*sagS*_D105A, and *ΔsagS*/pMJT1-*sagS_*L154A strains versus the *ΔsagS*/pMJT1-*bifA*-*sagS*, *ΔsagS*/pMJT1-*bifA*-*sagS*_H91A, and *ΔsagS*/pMJT1-*bifA*-*sagS*_L140A strains after 3 days of growth as determined using confocal images and subsequent COMSTAT analysis. All assays were repeated at least three times, with a minimum of 6 images being acquired. Error bars indicate standard deviation. ** (*P* < 0.01), *** (*P* < 0.001), and **** (*P* < 0.0001) indicate significantly different from the Δ*sagS*/pMJT1-*sagS* or Δ*sagS*/pMJT1-*bifA*-*sagS* strain. NS, not significantly different from the Δ*sagS*/pMJT1-*sagS* or Δ*sagS*/pMJT1-*bifA*-*sagS* strain. Download FIG S7, PDF file, 0.06 MB. Copyright © 2019 Dingemans et al. 2019 Dingemans et al. This content is distributed under the terms of the Creative Commons Attribution 4.0 International license.

To further explore the contribution of residue H91 to biofilm drug tolerance, we investigated the susceptibility phenotype of Δ*sagS* strains expressing *bifA-sagS*_H91A as well as *brlR* expression levels. For the latter, we made use of a GFP reporter assay to visualize P*brlR* promoter activity in *in vivo* biofilms grown in flow cells. Based on the knowledge that SagS contributes to biofilm tolerance via the indirect activation of BrlR (22, 27), as well as the fact that BrlR binds to its own promoter (28), we could test whether mutation of BifA-SagS affects *brlR* expression via this GFP reporter assay.

**FIG 8:**
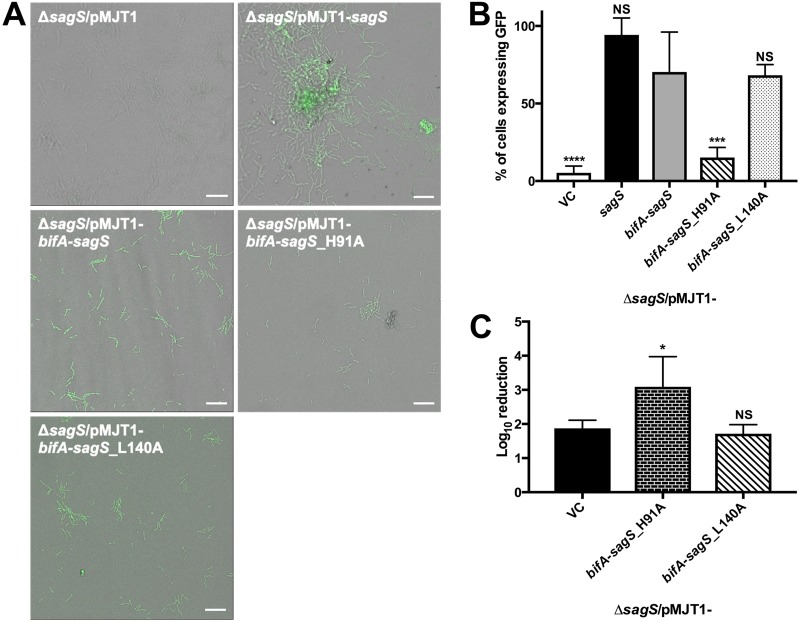
P*brlR* promoter activity and antibiotic susceptibility of the Δ*sagS*/pMJT1-*bifA-sagS* strain and its H91A and L140 variants. (A) Representative confocal images of GFP expression (indicative of P*brlR* promoter activity) by *ΔsagS* cells harboring the empty pMJT1 vector or expressing wild-type *sagS*, *bifA-sagS*, *bifA-sagS*_H91A, or *bifA-sagS*_L140A after 24 h of attachment in flow cells. White bars = 20 μm. (B) Quantification of the proportion of cells expressing GFP compared to the total population. Experiments were repeated at least two times, with a minimum of 3 images being acquired. Error bars denote standard deviation. (C) Tobramycin susceptibility of 2-day-old biofilms formed by *ΔsagS*/pMJT1-*bifA*-*sagS* cells compared to *ΔsagS*/pMJT1-*bifA*-*sagS*_H91A and *ΔsagS*/pMJT1-*bifA*-*sagS*_L140A cells. Viability was determined via CFU counts. Susceptibility is expressed as log_10_ reduction. Experiments were carried out in triplicate. Error bars denote standard deviation. * (*P* < 0.05), *** (*P* < 0.001), and **** (*P* < 0.0001) indicate significantly different from the Δ*sagS*/pMJT1-*bifA*-*sagS* strain. NS, not significantly different from the Δ*sagS*/pMJT1-*bifA*-*sagS* strain.

After 24 h of attachment in flow cells, P*brlR* promoter activity for the Δ*sagS*/pMJT1-*bifA*-*sagS* strain was intermediate between that observed for the Δ*sagS*/pMJT1 and Δ*sagS*/pMJT1-*sagS* strains (Fig. 8A), with 70.3% of the attached cells expressing green fluorescent protein (GFP) for the hybrid compared to 5.2% for the Δ*sagS*/pMJT1 strain and 94.3% for the mutant complemented with wild-type *sagS*, respectively (Fig. 8B). Furthermore, P*brlR* promoter activity was significantly reduced in the H91A variant (15.2% of cells expressing GFP) but not the L140A variant (68.2% of cells expressing GFP) compared to wild-type BifA-SagS (70.3% of cells expressing GFP) (Fig. 8A and B). These results are in agreement with a significant increase in tobramycin susceptibility for the H91A variant (3.1-log_10_ reduction) but not the L140A variant (1.7-log_10_ reduction) compared to wild-type BifA-SagS (1.9-log_10_ reduction) (Fig. 8C). Moreover, these findings are in agreement with SagS residue D105, but not residue L154, contributing to *brlR* expression and the antibiotic susceptibility phenotype by P. aeruginosa biofilms (23, 33).

## DISCUSSION

In P. aeruginosa, as well as many other bacteria, the transition from the planktonic to the biofilm lifestyle involves the complex interplay of two-component regulatory systems, c-di-GMP levels, and quorum sensing systems (QS), eventually leading to adhesion and the formation of an extracellular matrix composed of exopolysaccharides, extracellular DNA, and proteins (16, 17, 19, 21, 41, 42). However, the decision to switch to this sessile lifestyle is often triggered by an external stimulus that can be sensed through the sensory domain of histidine kinases, diguanylate cyclases (DGC), phosphodiesterases (PDEs), and methyl-accepting chemotaxis proteins among others (21, 43). The hybrid sensor kinase SagS plays an important role in the transition from the planktonic to the biofilm growth stage by regulating attachment/biofilm formation via a phosphorylation cascade that starts with the direct phosphotransfer to BfiS (20) and by rendering biofilm cells tolerant to antimicrobials via the indirect activation of BrlR (22, 27). In contrast to this, the phosphodiesterase BifA suppresses biofilm formation by decreasing the intracellular c-di-GMP pool (34). Although the sequences of the sensory domains of BifA and SagS show considerable divergence, with only a number of residues involved in the biofilm formation and antibiotic tolerance pathways being conserved between both proteins, here we show that they share great predicted structural similarity (Fig. 1). In an effort to start investigating cues sensed by bacteria that trigger a switch in the bacterial lifestyle, we investigated in this study whether the periplasmic sensory domains of BifA and SagS, despite their disparate functions in biofilm formation and drug tolerance, are functionally interchangeable. While we did not specifically attempt to identify the ligands that interact with the sensory domains of BifA and SagS, we made use of both domains to determine potential conservation in signal relay. More specifically, we tested whether the mechanisms leading to signal transduction are conserved between both sensory domains (i) by designing a hybrid protein in which the N-terminal sensory domain of SagS was replaced by that of BifA and (ii) by alanine substitution of residues previously found to be key to SagS regulation of biofilm formation or antibiotic tolerance. Interestingly, complementation of a Δ*sagS* mutant with the hybrid partially restored attachment, biofilm formation, and antibiotic tolerance to a level intermediate between mutant and wild-type SagS. Furthermore, alanine substitution of residues L140 and H91 in the BifA-SagS hybrid, corresponding to residues L154 and D105 in SagS, significantly impaired the biofilm formation and antibiotic tolerance pathways, respectively. These results indicate that the predicted structural similarity between BifA and SagS leads to a similar mechanism by which a stimulus is sensed and transduced to the output domain, despite differences in sequence conservation. The similarity in signal transduction was furthermore supported by the finding that expression of the *sagS*-*bifA* hybrid in the Δ*bifA* mutant almost completely restored attachment and biofilm formation to wild-type levels, while the NicD-SagS hybrid, which contains a sensory domain belonging to a different family of ligand-binding domains, was nonfunctional. Overall, our data indicated that the nature of the sensory domain is important for proper signal transduction and functionality of the cytoplasmic portion of SagS.

Previous studies have reported that despite the large variability at the sequence level, many sensory domains fall into discrete structural classes and use similar mechanisms to sense and transduce signals (39, 43, 44). For example, the periplasmic sensory domains of Escherichia coli DcuS and Vibrio cholerae DctB only show moderate sequence similarity, but both contain a characteristic PDC (PhoQ, DcuS, CitA) sensor domain fold of the Cache domain superfamily and display similar modes of C_4_-dicarboxylate ligand binding (45). The fact that the H91A and L140A mutations have an impact on the divergent pathways that lead to biofilm formation and antibiotic tolerance in the BifA-SagS hybrid may indicate that BifA and SagS transduce their perceived cues or signals in a similar manner to activate a complex regulatory network involving multiple molecular players enabling the decision to switch to the biofilm stage of growth. A complex regulatory network seems likely since SagS positively regulates biofilm formation and antibiotic tolerance, whereas BifA negatively impacts c-di-GMP levels via its phosphodiesterase activity. This raises the question of whether the conservation in signal transduction also means that both sensory domains sense similar cues through their structurally conserved ligand binding domain. If identical or highly similar cues are sensed by SagS and BifA, does this also mean that the presumed cue for both proteins is constitutively present under the growth conditions tested in this study or that multicopy expression of the corresponding genes overrides the native stimulation by their respective cues? The notion of two proteins sensing similar cues but having opposite effects on biofilm formation strongly suggests that SagS and BifA only function temporarily or that additional cofactors or protein interaction partners are required for SagS and BifA to transmit the perceived signal(s) to their cytoplasmic effector domains. Potential cofactors include divalent cations, as it has been shown that V. cholerae DctB was crystallized in complex with its ligand succinate and Ca^2+^ (45), while the KinD sensor kinase of Bacillus subtilis induces biofilm formation when sensing a combination of glycerol and Mn^2+^ (46). Furthermore, it is possible that the sensory domains of BifA and SagS harbor a site for protein-protein interaction(s). One example in which such protein interactions affect activity can be found in E. coli, in which the lipoprotein QseG, which is attached to the inner leaflet of the outer membrane, interacts with the periplasmic sensory domain of the sensor kinase QseE and affects the phosphorylation state of QseE as well as its cognate response regulator QseF in response to a signal deriving from the cell envelope (47). Furthermore, it was found that the ability of QseE to sense epinephrine depends on its interaction with QseG (47). Another example can be found in P. aeruginosa, where PilA directly interacts with PilJ via its periplasmic sensory domain to transduce mechanically induced conformational changes in stretched type IV pili (48). Since SagS (and BifA-SagS) regulates biofilm formation and antibiotic tolerance via independent signaling pathways through distinct residues in its sensory domain, it is likely that SagS interacts with multiple ligands and/or proteins. These interactions may not be limited to the periplasmic HmsP sensory domain but extend to the cytoplasmic portion of SagS to regulate the toggle switch functions of SagS.

In summary, this study has investigated whether signal sensing and transduction are conserved between the sensor kinase SagS and the phosphodiesterase BifA, based on the observation that their sensory domains appear structurally similar despite moderate sequence conservation. Our data indicate that replacing the sensory domain of SagS by that of BifA partially restores the biofilm formation and antibiotic tolerance pathways in a Δ*sagS* mutant, while substitution of the sensory domain of BifA by that of SagS almost completely restored attachment and biofilm formation to wild-type levels in a Δ*bifA* background. In addition, it was found that mutation of residues in BifA that were previously found to be crucial for SagS in regulating both pathways affects BifA signaling in a similar way. Based on these findings, we conclude that BifA and SagS likely deploy a similar mechanism of signal sensing and transduction.

## MATERIALS AND METHODS

### Structure prediction of the HmsP and HAMP domains of SagS and BifA-SagS.

The amino acid sequence of the isolated HmsP and HAMP domains of SagS and BifA were first submitted to the PHYREII Protein Fold Recognition Server (49) to obtain a prediction model of the structure of the HmsP domain. Dimer homology models for the HmsP and HAMP domains of BifA were generated using the software package Modeller (50) and PDB codes 4U64 (51) and 2L7H (52) as the templates. Default parameters were used during PHYREII- and Rosetta-based modeling. The resulting models were visualized using PyMOL.

### Bacterial strains, plasmids, and culture conditions.

All bacterial strains and plasmids used in this study are listed in Table 1. Overnight cultures were grown in Lennox broth (LB) at 37°C under shaking conditions (220 rpm). Antibiotics for plasmid selection or maintenance were used at the following concentrations: 60 μg/ml tetracycline and 250 μg/ml carbenicillin for P. aeruginosa and 100 μg/ml ampicillin for E. coli.

**TABLE 1:** List of strains and plasmids used in this study

Strain or plasmid	Relevant genotype or description	Source or reference
Strains		
Escherichia coli		
NEB 5-alpha Competent (high efficiency)	*fhuA2* (*argF-lacZ*)*U169 phoA glnV44* ϕ80 (*lacZ*)M15 *gyrA96 recA1* *relA1 endA1 thi-1 hsdR17*	New England Biolabs
Pseudomonas aeruginosa		
PAO1	Wild type	59
Δ*sagS*	PAO1 Δ*sagS* (PA2824)	20
PA14	Wild type	34
Δ*bifA*	PA14 Δ*bifA* (PA14_56790)	34

Plasmids		
pMJT1-*sagS*	C-terminal HA-tagged *sagS* cloned into pMJT1 at NheI/SacI; Amp^r^ (Carb^r^)	22
pMJT1-*bifA*-*sagS*	C-terminal HA-tagged *bifA*-*sagS* hybrid cloned into pMJT1 at NheI/SacI; Amp^r^ (Carb^r^)	This study
pMJT1-*nicD*-*sagS*	C-terminal HA-tagged *nicD*-*sagS* hybrid cloned into pMJT1 at NheI/SacI; Amp^r^ (Carb^r^)	This study
pMJT1-*bifA*	C-terminal HA-tagged *bifA* cloned into pMJT1 at NheI/SacI; Amp^r^ (Carb^r^)	This study
pMJT1-*sagS*-*bifA*	C-terminal HA-tagged *sagS*-*bifA* hybrid cloned into pMJT1 at NheI/SacI; Amp^r^ (Carb^r^)	This study
pMJT1-*bifA*-*sagS* _H91A	C-terminal HA-tagged *bifA*-*sagS* hybrid harboring an H91A substitution in pMJT1; Amp^r^ (Carb^r^)	This study
pMJT1-*bifA*-*sagS* _L140A	C-terminal HA-tagged *bifA*-*sagS* hybrid harboring an L140A substitution in pMJT1; Amp^r^ (Carb^r^)	This study
p*brlR*-*gfp*	pMini-CTX-*gfp* with 1–750 bp upstream of *brlR*, Tet^r^	28
pRK2013	Helper plasmid for triparental mating; *mob tra* Km^r^	60

### Strain construction.

To generate the *bifA*-*sagS* hybrid, the HmsP domain of *bifA* (nucleotides 1 to 744, corresponding to amino acid residues 1 to 248) and the HiskA and Rec domains of *sagS* (nucleotides 787 to 2358, corresponding to amino acid residues 263 to 786) were PCR amplified using the Q5 Hot-Start high-fidelity 2× master mix (New England Biolabs) and the bifA_HmsP_F/bifA_HmsP_R and bifA_sagS_HisKA_F/sagS_HisKA_R primers (Table 2), respectively. Likewise, the *nicD*-*sagS* hybrid was constructed by amplifying the sequence encoding the N-terminal sensory domain and first transmembrane helix of *nicD* (nucleotides 1 to 762, corresponding to amino acid residues 1 to 254) and the sequence encoding the HAMP, HiskA, and Rec domains of *sagS* (nucleotides 583 to 2358, corresponding to amino acid residues 195 to 786) using the nicD_F/nicD_R and nicD_sagS_HisKA_F/sagS_HisKA_R primers (Table 2). Additionally, a *sagS*-*bifA* hybrid was generated by PCR amplification of the HmsP domain of *sagS* in PA14 (nucleotides 1 to 786, corresponding to amino acid residues 1 to 262) and the GGDEF/EAL domains of *bifA* in PA14 (nucleotides 745 to 2061, corresponding to amino acid residues 249 to 687) using the sagS_PA14_F/sagS_PA14_R and bifA_PA14_F/bifA_PA14_R primers (Table 2). Finally, the bifA_HmsP_F and bifA_PA14_R primers were used to amplify the wild-type *bifA* gene in PA14 (Table 2). PCR products were purified using the Wizard SV gel and PCR clean-up system (Promega) and the pMJT1 plasmid was digested using the NheI-HF and SacI-HF restriction enzymes (New England Biolabs). Finally, the PCR fragments were assembled and cloned into the pMJT1 vector using the DNABuilder HiFi DNA assembly cloning kit (New England Biolabs). More specifically, the assembly reaction was performed for 15 min at 50°C using a vector/insert ratio of 1:2. This was accomplished by combining 0.02 pmol of digested vector with 0.04 pmol of both PCR products (or in the case of wild-type *bifA* only one PCR fragment). Finally, 2 μl of the assembly reaction was transformed into E. coli DH5α (New England Biolabs), and transformants were screened via PCR using the bifA_HmsP_F/sagS_HisKA_R, nicD_F/sagS_HisKA_R, sagS_PA14_F/bifA_PA14_R, or bifA_HmsP_F/bifA_PA14_R primers (Table 2). Plasmid DNA was purified from positive transformants and confirmed by sequencing. Plasmids were transferred into a Δ*sagS* (PAO1) or Δ*bifA* (PA14) mutant via electroporation (53).

**TABLE 2:** Oligonucleotides used in this study

Oligonucleotide	Sequence^*a*^
Cloning, sequencing, and PCR screening	
bifA_HmsP_F	TCCATACCCGTTTTTTTGGGCTAGC**TTG**AAACTGGACTCCCGACAC
bifA_HmsP_R	CCTCCAGGTAGCGCAGCAGGTTGTCCTC
bifA_sagS_HisKA_F	CCTGCTGCGCTACCTGGAGGAGCTGGAAAG
sagS_HisKA_R	CATGATTACGAATTCGAGCTC**CTA**AGCGTAGTCTGGGACGTCGTATGGGTAGTCGCTCGCGGTGAG
nicD_F	TCCATACCCGTTTTTTTGGGCTAGC**ATG**AGCAATTCCGACGTTTTC
nicD_R	TCAGCAGGGTGCTGGTGAAGAGGAACAG
nicD_sagS_HisKA_F	CTTCACCAGCACCCTGCTGACCAAGCCC
sagS_PA14_F	TCCATACCCGTTTTTTTGGGCTAGC**ATG**CTAGGCGGCAGAACC
sagS_PA14_R	ACTGGGAAATCTGGGTCAGGCGGTTCTC
bifA_PA14_F	CCTGACCCAGATTTCCCAGTACGACTTCCTCACC
bifA_PA14_R	CATGATTACGAATTCGAGCTC**TCA**AGCGTAGTCTGGGACGTCGTATGGGTAGGGCCGTTCGCTGCT
Pser-up	CGAGTGGTTTAAGGCAACGGTCTTGA
Pser-down	AGTTCGGCCTGGTGGAGCAACTCG

Site-directed mutagenesis	
H91A_F	GAGCATCGGCgcTCCCGGCGAGCCGATGC
H91A_R	GCCTGGCGCACCGCCTCG
L140A_F	CTACGGCGACgcGAAGATCACCCTG
L140A_R	TATTCGCTATAGGGCGGG

RT-qPCR	
mreB_F	CTTCATCAACAAGGTCCACGA
mreB_R	GCTCTTCGATCAGGAACACC
brlR_F	GTGGTGGGCATGGAATACTT
brlR_R	ATAGGAGACCTCGGGATCGT

a Start and stop codons are indicated in bold, and the sequence encoding the HA tag is underlined. The lowercase “gc” indicates the nucleotides that were changed, resulting in an alanine substitution.

Site-directed mutations in the *bifA*-*sagS* hybrid were generated using the Q5 site-directed mutagenesis kit (New England Biolabs) according to the manufacturer’s protocol and using the primers listed in Table 2. The respective pMJT1 plasmids harboring *bifA*-*sagS* with the site-directed mutations were sequenced and transferred into the Δ*sagS* (PAO1) mutants by electroporation (53).

### Attachment assays.

Overnight cultures of P. aeruginosa cells were diluted 100-fold in LB medium containing 250 μg/ml carbenicillin to an optical density at 600 nm (OD_600_) of 0.025, and 200 μl of the resulting dilution was added to each well of a 96-well plate followed by 24 h of incubation at 37°C with continuous shaking at 220 rpm, Next, 50 μl of a 0.1% (wt/vol) crystal violet solution was added to each well, and plates were incubated for 15 min at 37°C while shaking. Plates were washed three times with 200 μl of Nanopure water to remove nonattached cells and excess crystal violet and allowed to dry. Finally, the remaining crystal violet was resuspended in 200 μl of 95% ethanol, and the OD_570_ was determined. Blank values (LB medium alone) were subtracted, and data were normalized to the values obtained for the P. aeruginosa Δ*sagS* mutant overexpressing *bifA*-*sagS*.

### Biofilm formation.

For biofilm antibiotic susceptibility and RNA extraction, biofilms were grown in a continuous-flow reactor system with size 13 (1 m in length) Masterflex silicone tubing (Cole Parmer) at a flow rate of 0.1 ml/min, as previously described using 20-fold-diluted LB medium (3–5). For protein extraction, biofilms were grown in a continuous-flow reactor system with size 14 (1 m in length) Masterflex silicone tubing (Cole Parmer) at a flow rate of 0.2 ml/min using 20-fold-diluted LB medium. Carbenicillin at 10 μg/ml was added to the growth medium for plasmid maintenance. To visualize and quantify biofilm formation, biofilms were grown in 24-well plates in 5-fold-diluted LB medium containing 10 μg/ml carbenicillin, with the growth medium being exchanged every 12 h as previously described (23). Confocal laser scanning microscopy (CLSM) images were acquired using a Leica TCS SP5 confocal microscope (Leica Microsystems, Inc., Wetzlar, Germany) and the LIVE/DEAD BacLight bacterial viability kit (Life Technologies, Inc.). Quantitative analysis of the confocal laser scanning microscope images of 24-well plate-grown biofilms was performed using COMSTAT (54).

### Biofilm antibiotic susceptibility testing.

To determine susceptibility of biofilm cells to antibiotic treatment, biofilms were grown for 2 days using a continuous flow reactor. Next, biofilm cells in the continuous-flow reactor were exposed to tobramycin (150 μg/ml) for 1 h under flowing conditions. Upon completion of the treatment, the cells were harvested and homogenized to disrupt any aggregates and cell clusters. The homogenized cells were serially diluted, and up to 7 dilutions were plated onto LB agar. Following overnight incubation at 37°C, viability was determined via CFU counts; susceptibility is expressed as log_10_ reduction.

### Immunoblot analysis.

HA-tagged SagS, BifA-SagS, NicD-SagS, and SagS-BifA were assessed via SDS-PAGE and subsequent immunoblot analysis. Overnight cultures of P. aeruginosa were diluted 100-fold in LB medium containing 250 μg/ml carbenicillin, grown to late exponential phase, and induced with 1% arabinose for 2 h at 37°C with continuous shaking at 220 rpm. Next 1 ml of the culture was pelleted and resuspended in 250 μl of TE buffer (10 mM Tris-HCl [pH 8.0], 1 mM EDTA) containing 0.3 μg/ml phenylmethylsulfonyl fluoride (PMSF), followed by lysis via sonication. Finally, 15 μg (P. aeruginosa PAO1) or 30 μg (P. aeruginosa PA14) of total cell extract was loaded on an SDS-PAGE gel and analyzed via immunoblotting using anti-HA antibodies.

### Phosphoprotein enrichment and detection.

Phosphorylated proteins were enriched via metal oxide affinity chromatography (MOAC) essentially as described by Wolschin and colleagues (55). MOAC has been demonstrated by Krüger et al. to result in up to 20-fold enrichment of phosphoproteins and to approach 100% specificity (56). To determine phosphorylation of BfiS by BifA-SagS under biofilm conditions, a pJN-bfiS-V5/HisVI construct was introduced in each complemented Δ*sagS* mutant. Biofilms were grown in 20-fold-diluted LB containing 10 μg/ml carbenicillin, 2 μg/ml gentamicin, and 0.1% arabinose for 3 days under flowing conditions (0.2 ml/min), harvested by extrusion of the cell paste, and resuspended in 500 μl of TE buffer (10 mM Tris-HCl [pH 8.0], 1 mM EDTA) containing 0.3 μg/ml phenylmethylsulfonyl fluoride (PMSF), followed by lysis via sonication. Briefly, 750 μg of total cell extract (TCE) was incubated with MOAC incubation buffer (30 mM MES [morpholineethanesulfonic acid], 0.2 M potassium glutamate, 0.2 M sodium aspartate, 0.25% CHAPS {3-[(3-cholamidopropyl)-dimethylammonio]-1-propanesulfonate}, and 8 M urea) in a final volume of 1.5 ml and subsequently incubated for 30 min at 4°C in the presence of 80 mg of aluminum hydroxide. Unbound proteins were removed via six 1-min washes with 1.5 ml of incubation buffer at 16,000 × *g* and 4°C. Next, phosphoproteins were eluted from the slurry using 100 mM potassium pyrophosphate and 8 M urea, desalted by methanol-chloroform precipitation, and subsequently vacuum dried. Finally, samples were analyzed by SDS-PAGE, followed by the detection of BfiS-V5/HisVI by immunoblotting with anti-V5 antibodies as described above. Aliquots obtained prior to MOAC were used as loading controls.

### RNA isolation and qRT-PCR.

Biofilms grown in biofilm tube reactors were harvested by extrusion, with the cell paste being collected directly into 500 μl of RNAProtect bacterial reagent (Qiagen), followed by RNA extraction as previously described (33). Any remaining genomic DNA was removed using 1 μl of (2 U/μl) Turbo DNase (Ambion) for 30 min, and cDNA was prepared using the iScript Select cDNA synthesis kit (Bio-Rad) starting from 1 μg of total RNA. Reverse transcription-quantitative PCR (qRT-PCR) was performed using the Bio-Rad CFX Connect real-time PCR detection system and SsoAdvanced SYBR green supermix (Bio-Rad) with primers listed in Table 2. The *mreB* gene was used as a housekeeping gene. Fold changes were calculated using the Livak method (57). Melting curve analyses were performed to verify specific single-product amplification.

### GFP reporter assay to determine P*brlR* promoter activity.

To determine P*brlR* promoter activity, the P*brlR*-*gfp* construct, in which the *gfp* gene is cloned under the P*brlR* promoter in the pMini-CTX vector (Table 1), was introduced into the Δ*sagS* mutant strain via triparental mating, and proper integration into the chromosome was verified via PCR using Pser-up/Pser-down primers as previously described (28, 33). Next, the resulting Δ*sagS* mutant harboring the P*brlR*-*gfp* construct was complemented with either pMJT1, pMJT1-*sagS*, or pMJT1-*bifA*-*sagS* or its two site-directed variants pMJT1-*bifA*-*sagS* _H91A and pMJT1-*bifA*-*sagS* _L140A. To detect GFP reporter activity, the respective P. aeruginosa strains were grown in flow cells (in 20-fold-diluted LB medium supplemented with 10 μg/ml carbenicillin) and allowed to attach for 24 h since *gfp* expression has been found to be maximal at this point as previously reported (28). Confocal laser scanning microscopy (CLSM) images were acquired using a Leica TCS SP5 confocal microscope (Leica Microsystems, Inc., Wetzlar, Germany), and the proportion of cells expressing GFP was determined using ImageJ software (58).

### Statistical analysis.

Statistical differences between strains were determined using a one-way analysis of variance (ANOVA), followed by a Dunnett’s *post hoc* test using PrismV software (Graph Pad, La Jolla, CA, USA).
