# Supplementary material for: Signal Sensing and Transduction Are Conserved between the Periplasmic Sensory Domains of BifA and SagS
Source: mSphere. 2019 Jul 31;4(4):e00442-19. doi: 10.1128/mSphere.00442-19 (PMC6669338; doi:10.1128/mSphere.00442-19)
Supplement: FIG S1 [file mSphere.00442-19-sf001.pdf]

```
BifA-HmsP      1 --------------MKLDSRHSLSLKLLRVVLLAALAVGVVLSCAQIVFD     36
                               |.:...|.||.|...:.:|.|..:|.:||..|:..|
SagS-HmsP      1 MLGGRTSPRLIPAPMDIALTHRLSFKQASLTVLVAFILGTLLSLIQVGVD     50

BifA-HmsP     37 AYKAKQAVSSDAQRILAMVRDPSTQAVYSLDREMAMQVLEGLFQHEAVRQ     86
                 ......:::.:.:.:|.:..:|:.:..|::|.|:|.:::.||.:..||.:
SagS-HmsP     51 YASQDASINREVRALLDVSHNPAARIAYNIDAELAQELVLGLLRSPAVVR    100

BifA-HmsP     87 ASIGHPGEPMLAEKSRPLLDLPTRWLTDPILGQERTFSIRL-IGRPPYSE    135
                 |.|.......||..||...:...|.|:|.:.|.:|.:...| :...| .|
SagS-HmsP    101 AEIIDTSGLPLASASREPAESRLRPLSDFLFGHKRVYEDPLHVDHAP-GE    149

BifA-HmsP    136 YYGDLKITLDTAPYGENFVTTSEIIFISGILRALAMGLVLFLVYHWMLTK    185
                 ..|.|.:.:||..:|.:|:..:.|..:||.:|:|.:.|:|.::::.:|||
SagS-HmsP    150 ALGVLHLEIDTFVFGNDFLRRAGITLLSGFVRSLLLSLILLVLFYTLLTK    199

BifA-HmsP    186 PLSKIIEHLVSINPDRPSQHQLPLLKGHERNELGLWVTTANQLLASIESN    235
                 ||..:|:.|...:|..|::.:||..|||||:|:|:.|...|:.|..|...
SagS-HmsP    200 PLVSLIQALSGHDPRSPARMRLPCPKGHERDEIGVLVEVINRQLGRISVE    249

BifA-HmsP    236 SHLRREAEDNLLR    248
                 ...|||||:.|.:
SagS-HmsP    250 IEQRREAENRLTQ    262
```
