# Supplementary material for: Signal Sensing and Transduction Are Conserved between the Periplasmic Sensory Domains of BifA and SagS
Source: mSphere. 2019 Jul 31;4(4):e00442-19. doi: 10.1128/mSphere.00442-19 (PMC6669338; doi:10.1128/mSphere.00442-19)
Supplement: FIG S2 [file mSphere.00442-19-sf002.pdf]

```
PA14_SagS-HmsP     1 MLGGRTSPRLIPAPMDIALTHRLSFKQASLTVLVAFILGTLLSLIQVGVD     50
                     ||||||||||||||||||||||||||||||||||||||||||||||||||
PAO1_SagS-HmsP     1 MLGGRTSPRLIPAPMDIALTHRLSFKQASLTVLVAFILGTLLSLIQVGVD     50

PA14_SagS-HmsP    51 YASQDASINREVRALLDVSHNPAARIAYNIDAELAQELVLGLLRSPAVVR    100
                     ||||||||||||||||||||||||||||||||||||||||||||||||||
PAO1_SagS-HmsP    51 YASQDASINREVRALLDVSHNPAARIAYNIDAELAQELVLGLLRSPAVVR    100

PA14_SagS-HmsP   101 AEIIDTSGLPLASASREPAESRLRPLSDFLFGHKRVYEDPLHVDHAPGEA    150
                     ||||||||||||||||||||||||||||||||||||||||||||||||||
PAO1_SagS-HmsP   101 AEIIDTSGLPLASASREPAESRLRPLSDFLFGHKRVYEDPLHVDHAPGEA    150

PA14_SagS-HmsP   151 LGVLQLEIDTFVFGNDFLRRAGITLLSGFVRSLLLSLILLVLFYTLLTKP    200
                     ||||.|||||||||||||||||||||||||||||||||||||||||||||
PAO1_SagS-HmsP   151 LGVLHLEIDTFVFGNDFLRRAGITLLSGFVRSLLLSLILLVLFYTLLTKP    200

PA14_SagS-HmsP   201 LVSLIQALSGHDPRSPARMRLPCPKGHERDEIGVLVEVINRQLGRISVEI    250
                     ||||||||||||||||||||||||||||||||||||||||||||||||||
PAO1_SagS-HmsP   201 LVSLIQALSGHDPRSPARMRLPCPKGHERDEIGVLVEVINRQLGRISVEI    250

PA14_SagS-HmsP   251 EQRREAENRLTQ     262
                     ||||||||||||
PAO1_SagS-HmsP   251 EQRREAENRLTQ     262
```
