# Supplementary material for: Signal Sensing and Transduction Are Conserved between the Periplasmic Sensory Domains of BifA and SagS
Source: mSphere. 2019 Jul 31;4(4):e00442-19. doi: 10.1128/mSphere.00442-19 (PMC6669338; doi:10.1128/mSphere.00442-19)
Supplement: FIG S3 [file mSphere.00442-19-sf003.pdf]

```
PA14_BifA-HmsP     1 MKLDSRHSLSLKLLRVVLLAALAVGVVLSCAQIVFDAYKAKQAVSSDAQR     50
                     ||||||||||||||||||||||||||||||||||||||||||||||||||
PAO1_BifA-HmsP     1 MKLDSRHSLSLKLLRVVLLAALAVGVVLSCAQIVFDAYKAKQAVSSDAQR     50

PA14_BifA-HmsP    51 ILAMVRDPSTQAVYSLDREMAMQVLEGLFQHEAVRQASIGHPGEPMLAEK    100
                     ||||||||||||||||||||||||||||||||||||||||||||||||||
PAO1_BifA-HmsP    51 ILAMVRDPSTQAVYSLDREMAMQVLEGLFQHEAVRQASIGHPGEPMLAEK    100

PA14_BifA-HmsP   101 SRPLLDLPTRWLTDPILGQERTFSIRLIGRPPYSEYYGDLKITLDTAPYG    150
                     ||||||||||||||||||||||||||||||||||||||||||||||||||
PAO1_BifA-HmsP   101 SRPLLDLPTRWLTDPILGQERTFSIRLIGRPPYSEYYGDLKITLDTAPYG    150

PA14_BifA-HmsP   151 ENFVTTSEIIFISGILRALAMGLVLFLVYHWMLTKPLSKIIEHLVSINPD    200
                     ||||||||||||||||||||||||||||||||||||||||||||||||||
PAO1_BifA-HmsP   151 ENFVTTSEIIFISGILRALAMGLVLFLVYHWMLTKPLSKIIEHLVSINPD    200

PA14_BifA-HmsP   201 RPSQHQLPLLKGHERNELGLWVTTANQLLASIESNSHLRREAEDNLLR    248
                     ||||||||||||||||||||||||||||||||||||||||||||||||
PAO1_BifA-HmsP   201 RPSQHQLPLLKGHERNELGLWVTTANQLLASIESNSHLRREAEDNLLR    248
```
