# Supplementary figures and images for: Signal Sensing and Transduction Are Conserved between the Periplasmic Sensory Domains of BifA and SagS
Source: mSphere. 2019 Jul 31;4(4):e00442-19. doi: 10.1128/mSphere.00442-19 (PMC6669338; doi:10.1128/mSphere.00442-19)

**A**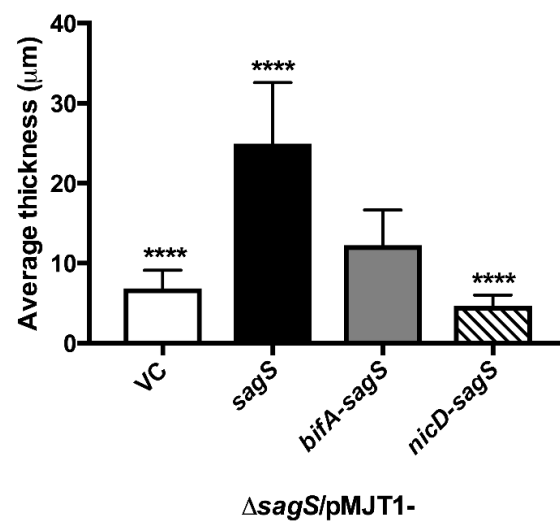**B**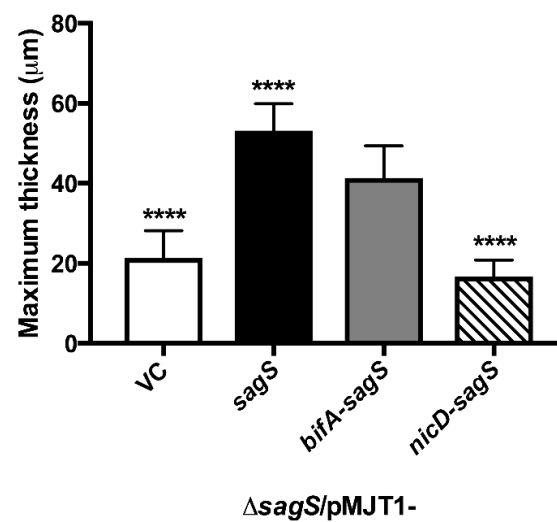

Supplement: FIG S4 [file mSphere.00442-19-sf004.pdf]

**A**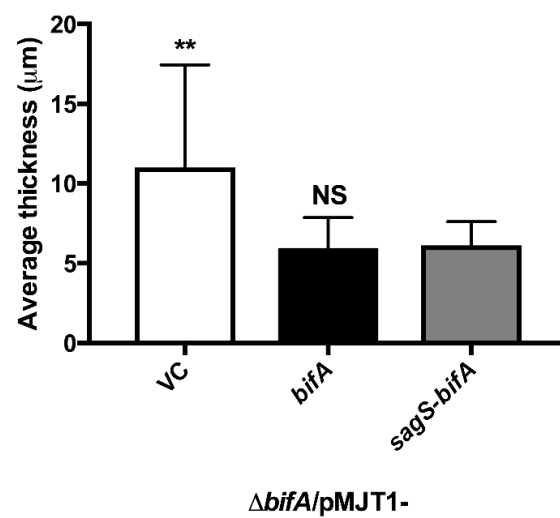**B**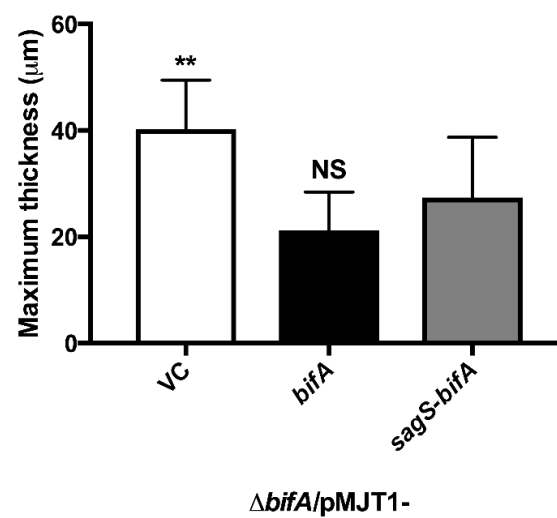

Supplement: FIG S5 [file mSphere.00442-19-sf005.pdf]

**A**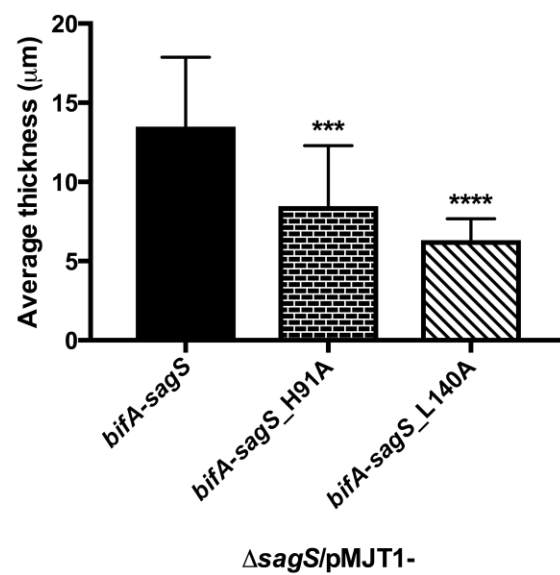**B**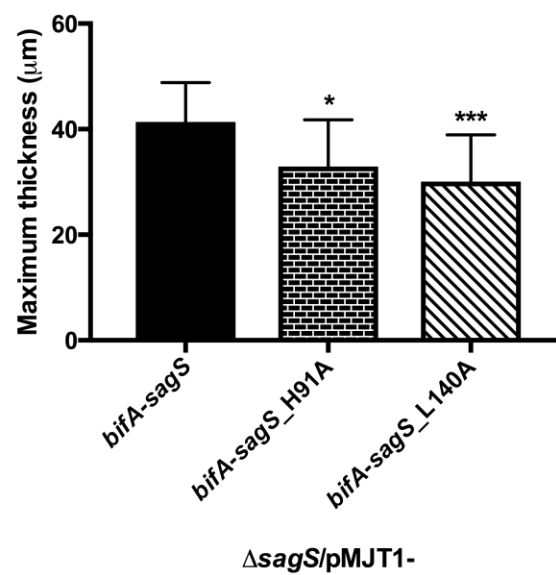

Supplement: FIG S6 [file mSphere.00442-19-sf006.pdf]

**A**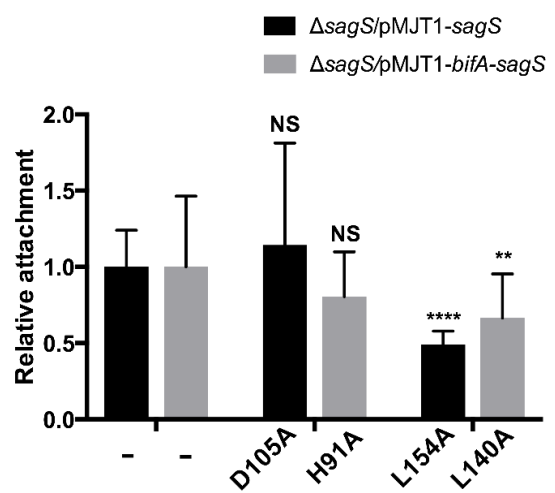**B**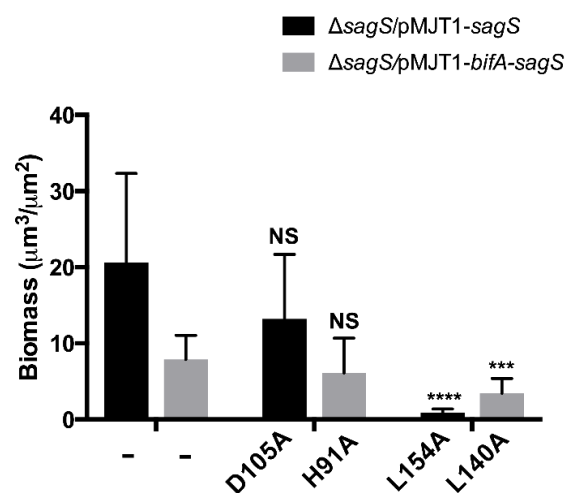

Supplement: FIG S7 [file mSphere.00442-19-sf007.pdf]
